# HER2-Low Status Does Not Affect Survival Outcomes of Patients with Metastatic Breast Cancer (MBC) Undergoing First-Line Treatment with Endocrine Therapy plus Palbociclib: Results of a Multicenter, Retrospective Cohort Study

**DOI:** 10.3390/cancers14204981

**Published:** 2022-10-11

**Authors:** Francesca Carlino, Anna Diana, Anna Ventriglia, Antonio Piccolo, Carmela Mocerino, Ferdinando Riccardi, Domenico Bilancia, Francesco Giotta, Giulio Antoniol, Vincenzo Famiglietti, Salvatore Feliciano, Rodolfo Cangiano, Lorenzo Lobianco, Benedetta Pellegrino, Ferdinando De Vita, Fortunato Ciardiello, Michele Orditura

**Affiliations:** 1 Department of Precision Medicine, Division of Medical Oncology, University of Campania Luigi Vanvitelli, 80131 Naples, Italy; 2 Medical Oncology Unit, Ospedale Ave Gratia Plena, San Felice a Cancello, 81027 Caserta, Italy; 3 Medical Oncology Unit, Ospedale del Mare, 80147 Naples, Italy; 4 Medical Oncology Unit, Ospedale Cardarelli, 80131 Naples, Italy; 5 Operating Unit, Medical Oncology, Hospital “Azienda Ospedaliera S. Carlo”, 85100 Potenza, Italy; 6 Medical Oncology Unit, IRCCS-Istituto Tumori “Giovanni Paolo II”, 70124 Bari, Italy; 7 Département de Génie Informatique et Génie Logiciel, 2500 Chemin de Ecole Polytechnique de Montréal, Montreal, QC H3T 1J4, Canada; 8 Medical Oncology Unit, Ospedale Ave Gratia Plena, Piedimonte Matese, 81016 Caserta, Italy; 9 Medical Oncology and Breast Unit, University Hospital of Parma, 43126 Parma, Italy

**Keywords:** breast cancer, cyclin-dependent kinase inhibitor 4 and 6, HER2-low, palbociclib

## Abstract

**Simple Summary:**

Breast cancers (BCs) with a HER2 immunohistochemical score of 1+ or 2+ with negative in situ hybridization are referred as HER2-low BCs. The knowledge about the biological and clinical characteristics of HER2-low BCs is still limited and controversial. Despite that new anti-HER2 antibody-drug conjugates (ADCs) have demonstrated significant activity in HER2-low BCs, no anti-HER2 agents are currently approved for this subgroup in Europe. Therefore, treatment for HER2-low BCs is determined by HR expression status. In this study, we aimed to investigate the prognostic significance of HER2-low status in HR+/HER2 negative (HER2-) metastatic BC (MBC) patients treated with endocrine therapy (ET) plus palbociclib as first line. HR+ MBC patients with HER2-low tumors who received first-line treatment with ET plus palbociclib show similar survival outcomes compared to those HER2-0 disease.

**Abstract:**

Background: Approximately 45–50% of breast cancers (BCs) have a HER2 immunohistochemical score of 1+ or 2+ with negative in situ hybridization, defining the “HER2-low BC” subtype. No anti-HER2 agents are currently approved for this subgroup in Europe, where treatment is still determined by HR expression status. In this study, we investigated the prognostic significance of HER2-low status in HR+/HER2- metastatic BC (MBC) patients treated with endocrine therapy (ET) plus palbociclib as first line. Methods: We conducted a retrospective study including 252 consecutive HR+/HER2- MBC patients who received first-line ET plus palbociclib at six Italian Oncology Units between March 2016 and June 2021. The chi-square test was used to assess differences in the distribution of clinical and pathological variables between the HER-0 and HER2-low subgroups. Survival outcomes, progression-free survival (PFS) and overall survival (OS), were calculated by the Kaplan–Meier method, and the log-rank test was performed to estimate the differences between the curves. Results: A total of 165 patients were included in the analysis: 94 (57%) and 71 (43%) patients had HER2-0 and HER2-low disease, respectively. The median age at treatment start was 64 years. No correlation between patients and tumor characteristics and HER2 status was found. Median PFS (mPFS) for the entire study cohort was 20 months (95% CI,18–25 months), while median OS (mOS) was not reached at the time of analysis. No statistically significant differences, in terms of PFS (*p* = 0.20) and OS (*p* = 0.1), were observed between HER2-low and HER2-0 subgroups. Conclusions: In our analysis, HR+ MBC patients with low HER2 expression who received first-line treatment with ET plus Palbociclib reported no statistically different survival outcomes compared to HER2-0 patients. Further prospective studies are needed to confirm the clinical role of HER2 expression level.

## 1. Introduction

Breast cancer (BC) is the most common malignancy and the leading cause of cancer morbidity among women worldwide [1]. BC presents a wide spectrum of heterogeneity in terms of gene expression, immunophenotypic features, response to treatment and clinical outcomes [2]. In daily clinical practice, therapeutic choice is still driven by the human epidermal growth factor receptor 2 (HER2) and hormone-receptor (HR) status, since gene expression profiling is not routinely available. Although tumors harboring HER2 positivity are characterized by a more aggressive behavior and poorer prognosis than other subtypes, the development of several anti-HER2 targeted agents has dramatically improved the survival outcomes of HER2 positive (HER2+) BC patients, both in early and advanced settings [3,4,5,6,7,8]. According to the dichotomic classification proposed by the 2018 American Society of Clinical Oncology (ASCO)/College of American Pathologists (CAP) guidelines, about 80% of breast tumors, lacking HER2 protein overexpression, are classified as HER2 negative (HER2-); within them, approximately 45–55% are characterized by HER2 immunohistochemistry (IHC) assay score of 1+ or 2+ with negative in situ hybridization (ISH), referred to as HER2-low BC [9,10]. In recent years, several studies have demonstrated the activity of the new anti-HER2 antibody-drug conjugates (ADCs) in both HER2+ and HER2-low BCs, leading researchers to shed new light on the latter type of tumors and to investigate whether they may represent a distinct subtype with specific behavior and prognosis [11]. The knowledge on the biological and clinical features of HER2-low BC is still limited and controversial [12]. Likewise, published studies evaluating the prognostic value of HER2-low expression have reported inconsistent and mixed results depending on disease stage and HR status (early vs. advanced, HR positive vs. triple negative breast cancer) [13,14,15,16,17,18]. Although ADCs have demonstrated to improve clinical outcomes of HER2-low tumors, this innovative strategy is not available yet in clinical practice for this subset of BC patients; therefore, the treatment choice is guided by HR expression status [7,8,19]. In detail, the combination of endocrine therapy (ET) plus cyclin-dependent kinase 4 and 6 inhibitors (CDK4/6i), palbociclib, ribociclib and abemaciclib, represents the mainstay of treatment for patients with HR+/HER2- metastatic disease, while for triple negative tumors, chemotherapy is still the standard of care, although promising drugs are emerging as novel therapeutic weapons. The advent of CDK4/6i has significantly changed the treatment paradigm with an impressive improvement in life expectancy for HR+/HER2- locally advanced or metastatic BC (MBC) patients. Despite this indisputable success, the knowledge of resistance mechanisms and the identification of potential biomarkers still represent an unmet clinical need. To this aim, several retrospective and prospective biomarker studies have been conducted [20]. Exploratory analyses of PALOMA-2 and 3 and MONALEESA trials have suggested a possible correlation between intrinsic subtypes and survival outcomes of MBC patients treated with CDK4/6i in addition to ET [21,22,23,24]. Furthermore, the bidirectional crosstalk between the HER and HR pathways as mechanisms of endocrine resistance could indicate a potential effect of low HER2 expression on CDK4/6i efficacy [25].

Recently, based on this assumption, Bao et al. conducted a retrospective analysis in patients treated with ET plus CDK4/6i, demonstrating a worse PFS in the HER2-low cohort compared to the HER2-0 subgroup [26]. These findings have led to further retrospective and subgroup analyses, which have reported opposite results [27]. Since this topic has raised a great deal of interest, in the present study, we evaluated the prognostic value of HER2 expression in a retrospective series of HR+/HER2- MBC patients treated with ET plus palbociclib as first line therapy.

## 2. Materials and Methods

### 2.1. Study Population

Data from 252 consecutive HR+/HER2- MBC patients who started treatment with palbociclib plus ET (aromatase inhibitors or fulvestrant) as first line treatment between March 2016 and June 2021 at six Medical Oncology Units were registered in a comprehensive database. We excluded patients for whom the date of diagnosis or metastatic relapse was unknown or in cases for whom HR and HER2 status or treatments were not documented. A total of 165 patients with HR+/HER2- MBC treated with ET plus palbociclib were eligible for the final analysis. Medical and pathology reports were reviewed for the following clinicopathological characteristics: age, histological type, tumor size, nodal involvement, grade of differentiation, HR and HER2 expression status, Ki67 index, type of treatments, date of diagnosis, date of relapse, and sites of metastases. A radiological assessment with total body computed tomography (CT) was performed at intervals of 3–4 months as recommended by guidelines and evaluated according to the criteria of Response Evaluation Criteria in Solid tumors (RECIST) 1.1.

This study was approved by the University of Campania Luigi Vanvitelli Ethic Committee (ID number: 15.03-20220005385*, 17 February 2022). All patients signed written informed consent form.

### 2.2. Definitions of Biomarker (ER, PR, Ki67 and HER 2)

Estrogen receptor (ER), progesterone receptor (PR) and Ki67 were assessed by IHC, and HER2 was assessed by IHC and/or ISH in primary tumor sample. When available, biomarkers obtained from the latest biopsy specimen were also collected. Biomarker positivity was detected and quantified as the percentage between immune-positive tumor cells and the total number of tumor cells and classified according to the St. Gallen and ASCO-CAP guidelines [28,29,30]. In particular, tumors were considered ER positive and/or PR positive when ≥1% of tumor cells demonstrate positive nuclear staining by IHC. Ki67 and PR were analyzed both as continuous and dichotomized variables (high vs. low). The cutoff for both variables was set at ≥20% and <20% of positively stained cells, for high and low, respectively [31,32]. HER2 was assessed using the HercepTest (DAKO Corporation), which uses the 0–3+ recommended scale to measure the percentage of immunoreactive neoplastic cells defined according to the intensity and completeness of membrane staining. A score of 3+ was considered HER2 positive. In cases of equivocal HER2 immunostaining (2+), ISH methodologies were applied [33]. Among HER2-negative tumors, HER2-low is referred to those with IHC score 1+ or 2+ and negative results on ISH [9]. There was no central review of biomarkers, but the pathological evaluations were performed by accredited anatomic pathology units, which ensure high rigor in methods and procedures in line with the best international standards.

### 2.3. Statistical Analysis

The primary study aim was to evaluate the survival outcomes, progression-free survival (PFS) and overall survival (OS) in a real-life cohort of HR+/HER2- BC patients who received ET (AI or fulvestrant) plus palbociclib as first line for metastatic disease categorized according to HER2 expression status as follows: HR+/HER2-0 or HR+/HER2-low. For descriptive analysis, percentages were used for categorical variables, and medians and ranges were used for continuous variables. PFS was the time from the beginning of treatment until disease progression or worsening. OS was defined as the time from treatment start to death from any cause. Patients who were alive at the last follow-up without recurrence or lost during follow-up and patients who had died without recurrence were censored at the date of the last recorded visit and the date of death, respectively. The median follow-up period was evaluated by the reverse Kaplan–Meier (KM) method. Chi square test was used to assess the differences in the distribution of clinical and pathological variables between the HER-0 and HER2-low subgroups. The survival curves were created using the KM method. The log-rank test was used to compare the differences among the curves [34]. Unadjusted hazard ratios (HRs) and univariate PFS probabilities were calculated using simple Cox proportional hazard regression models. Adjusted HRs for HER2 status were estimated by multivariate regression analysis with factors found to be statistically significantly associated with PFS in the univariate analyses using a *p* value threshold of 0.05. Similar analyses were performed for OS. All statistical analyses were performed using the R statistical computing environment release 4.1.2 on an Apple MacBook Pro M1 Max. In all computations, significance was assigned at *p* value of less than 0.05.

## 3. Results

### 3.1. Patients and Tumor Characteristics

A total of 165 patients were included in the analysis of who 94 (57%) had HER2-0 and 71 (43%) had HER2-low disease. Median age at treatment start was 64 years (34–88 years). Ductal carcinoma was the most common histological type found in 138 tumors (more than 83%); 112 (68%) and 131 (79%) cases displayed high (≥20%) Ki67 and PR levels, respectively. The majority of patients (64%, *n* = 106) had recurrent disease, while 59 patients (36%) presented with de novo metastatic breast cancer. Adjuvant ET was administered to 102 (62%) patients according to international guidelines. Among the 63 (38%) patients who did not receive adjuvant endocrine therapy, 59 were de novo metastatic and 4 relapsed during adjuvant chemotherapy. Regarding metastatic tumor burden, 63 (38%) and 102 (62%) patients had visceral and non-visceral disease, respectively. About two-thirds of the entire study cohort (112 patients) was AI sensitive (represented by patients who never received AI in early BC stage, or those who relapsed ≥12 months after completing adjuvant AI-based ET, or those who have been diagnosed with de novo stage IV BC), while 32% (53 patients) was AI-resistant (including patients who have been relapsed during adjuvant AI, or <12 months after its completion). Palbociclib was prescribed in combination with aromatase inhibitors or fulvestrant for AI-sensitive and AI-resistant patients, respectively. Key patient characteristics are presented in Table 1. No correlation between patients and tumor characteristics and HER2 status was found.

### 3.2. Survival Analysis

The median follow-up was 31 months (27.4–34.1 months) using 31 January 2022 as the data cut off. During this timeframe, progression of disease was registered in 105 patients (64%), of who 58 (55%) and 47 (45%) with HER2-0 and HER2-low tumors, respectively. Death for any cause occurred in 40 (38%) patients including 19 and 21 patients with HER2-0 and HER2-low tumors, respectively. The median PFS (mPFS) for the entire study cohort was 20 months (95% CI, 18–25 months). More than half of the study population was still alive at the time of the analysis; thus, median OS (mOS) could not be computed. Disease outcomes, in terms of PFS and OS, were compared between HER2-0 and HER2-low patients. No statistically significant differences, for each survival variable, were observed between the two subgroups. The mPFS for HER2-low was 19 months (95% CI, 14–21 months) compared with 23 months (95%CI, 18–27 months) for HER2-zero tumors (*p* = 0.20). Although median OS was not reached, the survival probabilities at 24, 36 and 48 months for HER2-0 and HER2-low patients were 86% and 80%, 76% and 60%, 62% and 51%, respectively (*p* = 0.1). (Table 2).

**Figure 1:**
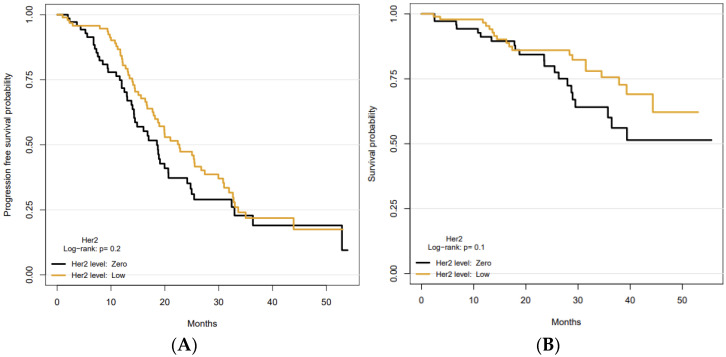
Kaplan–Meier curves demonstrating (**A**) progression-free survival, and (**B**) overall survival, according to HER2 status.

Kaplan–Meier curves for PFS and OS are shown in Figure 1.

In the univariate analysis for PFS, visceral disease and AI resistance were significantly associated with shorter PFS (*p* = 0.0041 and *p* = 0.05, respectively) while HER2-low expression seemed to be related to slightly, but not statistically significant, worse PFS (*p* = 0.18).

Results from multivariate Cox proportional hazard models, confirmed that AI resistance and visceral disease were significantly associated with poorer outcome (HR: 1.64; 95% CI, 1.09–2.45; *p* = 0.016 and HR: 1.92; 95% CI, 1.28–2.87; *p* = 0.001) (Table 3).

Univariate analysis for OS revealed that low PR value and visceral disease were clinical parameters significantly associated with a worse OS. The multivariable analysis confirmed that low PR levels and visceral disease were independently associated with shorter OS (HR: 2.43; 95% CI, 1.24–4.74; *p* < 0.001 and HR: 4.19; 95% CI, 2.17–8.08, *p* < 0.001) (Table 4).

## 4. Discussion

In HR+/HER2- MBC, CDK4/6i are now considered the standard of care, in combination with ET, as first- or second-line systemic treatment for both endocrine-sensitive and endocrine-resistant patients because of meaningful improvement in clinical outcomes [35,36,37,38,39,40]. Despite the excellent results reported in the randomized trials, in clinical practice, a wide heterogeneity in treatment response is observed between patients due to primary or acquired resistance. In our study population, the median PFS was 20 months, and although the direct comparison of clinical outcomes data between real world and randomized trials is not statistically appropriate, the shorter PFS of CDK4/6i in clinical practice suggests that a proper patients’ selection might be a determinant for treatment efficacy. Therefore, in the absence of validated biomarkers, key questions about their optimal use still remain open, including whether all patients with HR+ MBC should receive a CDK4/6i. Mechanisms of resistance to these agents are multifactorial, but biomarkers with the ability to recognize early relapsers, or to predict the beneficial effect of CDK4/6i, are still to be identified. In order to address this issue, several genomic and retrospective analyses have been performed, and many others are still ongoing. In particular, research efforts are focusing on circulating biomarkers, given their many advantages in terms of ease of application and reproducibility at different time points that can capture spatial and temporal heterogeneity [41,42]. Retrospective analyses of large, randomized trials, PALOMA-2 and 3 and MONALEESA, have suggested a correlation between intrinsic subtype and efficacy of palbociclib and ribociclib in HR+/HER2- ABC treated with endocrine-based strategies. In particular, a joint retrospective analysis of PALOMA-2 and PALOMA-3 clinical trials showed an absolute advantage of palbociclib in Luminal A and Luminal B tumors defined using The EdgeSeq Oncology Biomarker Panel on the collected FFPE samples [43]. In the retrospective exploratory analysis of MONALEESA trials, evaluating clinical outcomes of intrinsic subtypes defined using NanoString technologies, all breast cancers, except those with basal-like genomic features, gained a consistent advantage in terms of PFS and OS with ribociclib [22]. Based on these findings, we hypothesized that the surrogate BC subtype, mirroring specific IHC features and clinical behaviors, could also have particular influence on survival outcomes in a metastatic setting. In this context, HER2-low BC is emerging as a potential distinct entity among the heterogenous population of HER2-tumors, comprising a non-negligible proportion of HR+ BC. Some reports in early stage settings showed that patients with HER2-low expression were more often associated with worse clinical features such as lymph node positive, poorly differentiated tumor grading and higher proliferation index [13,16]. As a result of these differences in tumor characteristics, a probable effect of HER2-low expression on clinical outcomes has been hypothesized. Studies focused on the prognostic value of HER2 expression level are limited, and the available results are controversial, highly depending on HR status and disease setting [12,13,15,16]. In particular, some retrospective data support a possible negative prognostic impact of HER2-low status in early settings, since higher pCR rates were registered among HER-0 patients treated with neoadjuvant chemotherapy [14,15,16]. However, data extrapolated from early stage cannot be translated to the metastatic setting due to the different genetic backgrounds that influence the clinical validity of the prognostic factors in these two contexts. In particular, an exploratory OS analysis including 1304 patients with ABC extrapolated from two datasets did not demonstrate statistically significant survival differences between the HER2-low and HER2-0 groups (*p* = 0.787) regardless of HR status [13]. Similar conclusions were drawn by Agostinetto and colleagues who compared survival outcomes between HER2-low and non-HER-low BC [12]. The Austrian Study Group of Medical Tumor Therapy (AGMT) analyzed data of 1729 patients, derived from a comprehensive metastatic BC registry including a widely heterogeneous population unselected for HR status, type or line of treatment showing that low HER2- expression has no impact on prognosis of metastatic BC [44]. Similarly, the analysis of data collected in the PRAEGNANT registry did not demonstrate the validity of a low level of HER2 to discriminate different prognostic groups among ABC patients with either HR+ or TNBC [45]. A recent retrospective analysis focused on 106 women with ABC treated with palbociclib or ribociclib plus ET as first or second line showed a potential impact of low HER2 expression on survival outcomes. This specific biological behavior could be driven by a bidirectional crosstalk between the ER and the HER2/HER3 axis, leading to ET resistance mechanisms [25]. Subsequently, Tarantino et al. performed a subgroup analysis including only patients receiving first-line ET (AI or fulvestrant) plus a CDK4/6 inhibitor (palbociclib, ribociclib or abemaciclib), demonstrating that treatment outcome (PFS1) was not influenced by HER2- expression [27]. The unresolved “dilemma” of the prognostic value of HER2- expression prompted our research to investigate whether HER2 expression in metastatic HR-positive, HER2-negative disease could have an impact on clinical outcomes of patients treated with ET plus palbociclib as first line. In the present study, including only patients with HR+ MBC, HER2-low expression was found in 43% of the participants, in line with other large studies that reported rates ranging from 31% to 64% [46,47]. In our study cohort, the survival analyses revealed a mPFS of 19 and 23 months in HER2-low and HER2-0, respectively, and the survival probabilities at 24, 36 and 48 months were slightly better for HER2-0 in respect to HER2-low. These differences in survival outcomes, although too small to draw conclusions, could be attributed to a higher likelihood of developing endocrine resistance in patients with lower HER2 expression. Overall, these results are intriguing, but we are aware that our data should be interpreted with caution and cannot be generalized due to the retrospective nature of the design and small sample size examined. Overall, these data highlight the high prevalence of this subtype, strengthening that the prognostic value of HER2-low should be reconsidered and further investigated in light of new potential treatment strategies. In this context, a new generation of anti-HER2 agents, represented by ADCs, have recently proven clinical activity with an acceptable safety profile in HER2-low disease [11,48]. Based on DESTINY-Breast04 results, trastuzumab deruxtecan (T-DXd), an ADC in which the trastuzumab antitumor properties are associated with a potent cytotoxic payload, was approved in the United States as the first HER2-directed therapy for patients with HER2-low MBC. Since signaling interactions between ER and HER family receptors are well-known endocrine resistance mechanisms, co-targeting of the HER2 and ER pathways with T-DXd and ET (anastrazole or fulvestrant), respectively, is being investigated in the phase IB DESTINY-Breast08 [49]. In this direction, zenocutuzumab, a HER2-HER3 bispecific antibody combined with ET, has demonstrated encouraging antitumor effects in xenograft models, opening the window to a new possible chemotherapy-free approach for patients with endocrine-resistant HER2-low BC [50]. Clearly, our study presents limitations that require attention and restrict the validity of the conclusions drawn. In addition to the above-mentioned limits (the small sample size, the retrospective design), technical issues related to the HER2 status evaluation method cannot be neglected. In particular, HER2 evaluation accuracy and reproducibility could be affected by no standardized pre-analytical and post-analytical processes and by the lack of a central confirmation of pathological assessment. Future studies might benefit from central assessment of HER2 to ensure standardized scoring within the low-level range, as well as from a molecular profile or an assessment by mRNA quantification to generate a more refined biomarker to define low HER2-expressing tumors.

## 5. Conclusions

In conclusion, our exploratory analysis suggests that low HER2 expression does not affect survival outcome in patients with MBC undergoing first-line treatment with ET plus palbociclib. Further prospective studies are needed to confirm the clinical implication of HER2 expression level, especially in view of the availability of new targeted agents and/or treatment combination strategies that could be incorporated into a therapeutic sequence.

## Figures and Tables

**Table 1:** Association of HER2 status with clinical and pathological characteristics and the derived Chi square test *p* value.

Characteristics	HER2-0 (94)	HER2-Low (71)	*p* Value
Age, years			
<65	50 (53%)	40 (56%)	0.80
≥65	44 (47%)	31 (44%)	
Ki67 index %			
High ≥ 20%	59 (63%)	53 (75%)	0.14
Low < 20%	35 (37%)	18 (25%)	
Progesterone Receptor			
Low < 20%	16 (17%)	18 (25%)	0.26
High ≥ 20%	78 (83%)	53 (75%)	
Estrogen Receptor			
Low (1–9%)	1	1	0.88
Moderate (10–49%)	6	6	
High (50–95%)	87	64	
Grading			
Grade 1	12	4	0.32
Grade 2	50	43	
Grade 3	32	24	
Adjuvant Endocrine Therapy			
Tamoxifene	12 (20%)	13 (31%)	0.58
AI	40 (66,7%)	24 (57.1%)	
Tam + AI	8 (13.3%)	5 (11.9%)	
Not done	34 (36.2%)	29 (40.8%)	
Histologic type			
Ductal	78 (83%)	60 (85%)	0.90
Lobular	16 (17%)	11 (15%)	
Site of metastases			
Visceral	35 (37%)	28 (39%)	0.89
Non visceral	59 (63%)	43 (61%)	
AI sensitivity			
AI sensitive	59 (63%)	53 (75%)	0.15
AI resistant	35 (37%)	18 (25%)	
M status at diagnosis			
M0	61 (66%)	45 (63%)	0.97
M1	33 (34%)	26 (37%)	

AI, aromatase inhibitors; Tam, tamoxifene.

**Table 2:** Survival probability at 24, 36 and 48 months.

Months	Entire Population		Her2-0		Her2-Low	
	Survival Probability	95% CI	Survival Probability	95% CI	Survival Probability	95% CI
24	83%	77–88	86%	78–94	80%	70–80
36	69%	60–78	76%	65–87	60%	45–75
48	59%	47–70	62%	45–80	51%	34–69

CI, confidence interval.

**Table 3:** Prognostic variables for progression free survival in univariate analysis and multivariate analysis.

	Univariate Analysis		Multivariate Analysis	
Variables	HR (95% CI for HR)	*p* Value	HR (95% CI for HR)	*p* Value
HER2-0 vs. HER2-low	0.77 (0.52–1.1)	0.18		
PR < 20% vs. PR ≥ 20%	1.5 (0.93–2.3)	0.096		
M1 vs. M0	1.1 (0.71–1.6)	0.77		
Ki67 < 20% vs. Ki67 ≥ 20%	1 (0.69–1.6)	0.85		
Age ≥ 65 years vs. <65 years	1.2 (0.79–1.7)	0.45		
AI-resistant vs. AI-Sensitive	1.5 (1–2.2)	**0.05**	1.64 (1.09–2.45)	**0.016**
Visceral vs. non-visceral disease	1.8 (1.2–2.7)	**0.0041**	1.92 (1.28–2.87)	**0.001**

HR, hazard ratio; CI, confidence interval; PR, progesterone receptor; AI, aromatase inhibitor. Significant values are indicated in bold.

**Table 4:** Prognostic variables for overall survival in univariate and multivariate analysis.

	Univariate Analysis		Multivariate Analysis	
Variables	HR (95% CI for HR)	*p* Value	HR (95% CI for HR)	*p* Value
HER2 0 vs. HER2 low	0.62 (0.32–1.16)	0.13		
PR < 20% vs. PR ≥ 20%	2.4 (1.2–4.6)	**0.011**	2.43 (1.24–4.74)	**<0.001**
M1 vs. M0	1.29 (0.69–2.41)	0.42		
Ki67 < 20% vs. Ki67 ≥ 20%	0.75 (0.37–1.52)	0.44		
Age ≥ 65 vs. <65	1.24 (0.66–2.33)	0.48		
AI-resistant vs. AI-sensitive	0.95 (0.48–1.87)	0.89		
Visceral vs. non-visceral disease	4.12 (2.14–7.95)	**<0.001**	4.19 (2.17–8.08)	**<0.001**

HR, hazard ratio; CI, confidence interval; PR, progesterone receptor; AI, aromatase inhibitor. Significant values are indicated in bold.

## Data Availability

The data that support the findings of this study are available on request from the corresponding author.

## References

[1] https://www.cancer.gov/about-cancer/understanding/statistics.

[2] Polyak K. (2007). Breast cancer: Origins and evolution. J. Clin. Investig..

[3] Slamon D.J., Clark G.M., Wong S.G., Levin W.J., Ullrich A., McGuire W.L. (1987). Human breast cancer: Correlation of relapse and survival with amplification of the HER-2/neu oncogene. Science.

[4] Seshadri R., Firgaira F.A., Horsfall D.J., McCaul K., Setlur V., Kitchen P. (1993). Clinical significance of HER-2/neu oncogene amplification in primary breast cancer. The South Australian Breast Cancer Study Group. J. Clin. Oncol..

[5] Pondé N., Brandão M., El-Hachem G., Werbrouck E., Piccart M. (2018). Treatment of advanced HER2-positive breast cancer: 2018 and beyond. Cancer Treat. Rev..

[6] Riecke K., Witzel I. (2020). Targeting the Human Epidermal Growth Factor Receptor Family in Breast Cancer beyond HER2. Breast Care.

[7] Fehrenbacher L., Cecchini R.S., Geyer C.E., Rastogi P., Costantino J.P., Atkins J.N., Crown J.P., Polikoff J., Boileau J.F., Provencher L. (2020). NSABP B-47/NRG Oncology Phase III Randomized Trial Comparing Adjuvant Chemotherapy with or Without Trastuzumab in High-Risk Invasive Breast Cancer Negative for HER2 by FISH and With IHC 1+ or 2+. J. Clin. Oncol..

[8] Gianni L., Lladó A., Bianchi G., Cortes J., Kellokumpu-Lehtinen P.L., Cameron D.A., Miles D., Salvagni S., Wardley A., Goeminne J.C. (2010). Open-label, phase II, multicenter, randomized study of the efficacy and safety of two dose levels of Pertuzumab, a human epidermal growth factor receptor 2 dimerization inhibitor, in patients with human epidermal growth factor receptor 2-negative metastatic breast cancer. J. Clin. Oncol..

[9] Tarantino P., Hamilton E., Tolaney S.M., Cortes J., Morganti S., Ferraro E., Marra A., Viale G., Trapani D., Cardoso F. (2020). HER2-Low Breast Cancer: Pathological and Clinical Landscape. J. Clin. Oncol..

[10] Marchiò C., Annaratone L., Marques A., Casorzo L., Berrino E., Sapino A. (2021). Evolving concepts in HER2 evaluation in breast cancer: Heterogeneity, HER2-low carcinomas and beyond. Semin. Cancer Biol..

[11] Modi S., Jacot W., Yamashita T., Sohn J., Vidal M., Tokunaga E., Tsurutani J., Ueno N.T., Prat A., Chae Y.S. (2022). Trastuzumab Deruxtecan in Previously Treated HER2-Low Advanced Breast Cancer. N. Engl. J. Med..

[12] Agostinetto E., Rediti M., Fimereli D., Debien V., Piccart M., Aftimos P., Sotiriou C., de Azambuja E. (2021). HER2-Low Breast Cancer: Molecular Characteristics and Prognosis. Cancers.

[13] Schettini F., Chic N., Brasó-Maristany F., Paré L., Pascual T., Conte B., Martínez-Sáez O., Adamo B., Vidal M., Barnadas E. (2021). Clinical, pathological, and PAM50 gene expression features of HER2-low breast cancer. NPJ Breast Cancer.

[14] Denkert C., Seither F., Schneeweiss A., Link T., Blohmer J.U., Just M., Wimberger P., Forberger A., Tesch H., Jackisch C. (2021). Clinical and molecular characteristics of HER2-low-positive breast cancer: Pooled analysis of individual patient data from four prospective, neoadjuvant clinical trials. Lancet Oncol..

[15] Rossi V., Sarotto I., Maggiorotto F., Berchialla P., Kubatzki F., Tomasi N., Redana S., Martinello R., Valabrega G., Aglietta M. (2012). Moderate immunohistochemical expression of HER-2 (2+) without HER-2 gene amplification is a negative prognostic factor in early breast cancer. Oncologist.

[16] Eggemann H., Ignatov T., Burger E., Kantelhardt E.J., Fettke F., Thomssen C., Costa S.D., Ignatov A. (2015). Moderate HER2 expression as a prognostic factor in hormone receptor positive breast cancer. Endocr. Relat. Cancer.

[17] Gilcrease M.Z., Woodward W.A., Nicolas M.M., Corley L.J., Fuller G.N., Esteva F.J., Tucker S.L., Buchholz T.A. (2009). Even low-level HER2 expression may be associated with worse outcome in node-positive breast cancer. Am. J. Surg. Pathol..

[18] Poulakaki N., Makris G.M., Battista M.J., Böhm D., Petraki K., Bafaloukos D., Sergentanis T.N., Siristatidis C., Chrelias C., Papantoniou N. (2016). Hormonal receptor status, Ki-67 and HER2 expression: Prognostic value in the recurrence of ductal carcinoma in situ of the breast?. Breast.

[19] Burris H.A., Rugo H.S., Vukelja S.J., Vogel C.L., Borson R.A., Limentani S., Tan-Chiu E., Krop I.E., Michaelson R.A., Girish S. (2011). Phase II study of the antibody drug conjugate trastuzumab-DM1 for the treatment of human epidermal growth factor receptor 2 (HER2)-positive breast cancer after prior HER2-directed therapy. J. Clin. Oncol..

[20] Asghar U.S., Kanani R., Roylance R., Mittnacht S. (2022). Systematic Review of Molecular Biomarkers Predictive of Resistance to CDK4/6 Inhibition in Metastatic Breast Cancer. JCO Precis. Oncol..

[21] Turner N.C., Liu Y., Zhu Z., Loi S., Colleoni M., Loibl S., DeMichele A., Harbeck N., André F., Bayar M.A. (2019). Cyclin E1 Expression and Palbociclib Efficacy in Previously Treated Hormone Receptor-Positive Metastatic Breast Cancer. J. Clin. Oncol..

[22] Prat A., Chaudhury A., Solovieff N., Paré L., Martinez D., Chic N., Martínez-Sáez O., Brasó-Maristany F., Lteif A., Taran T. (2021). Correlative Biomarker Analysis of Intrinsic Subtypes and Efficacy Across the MONALEESA Phase III Studies. J. Clin. Oncol..

[23] Finn R.S., Liu Y., Zhu Z., Martin M., Rugo H.S., Diéras V., Im S.A., Gelmon K.A., Harbeck N., Lu D.R. (2020). Biomarker Analyses of Response to Cyclin-Dependent Kinase 4/6 Inhibition and Endocrine Therapy in Women with Treatment-Naïve Metastatic Breast Cancer. Clin. Cancer Res..

[24] Prat A., Parker J.S. (2020). Standardized versus research-based PAM50 intrinsic subtyping of breast cancer. Clin. Transl. Oncol..

[25] Giuliano M., Trivedi M.V., Schiff R. (2013). Bidirectional Crosstalk between the Estrogen Receptor and Human Epidermal Growth Factor Receptor 2 Signaling Pathways in Breast Cancer: Molecular Basis and Clinical Implications. Breast Care.

[26] Bao K.K.H., Sutanto L., Tse S.S.W., Cheung K.M., Chan J.C.H. (2021). The Association of ERBB2-Low Expression with the Efficacy of Cyclin-Dependent Kinase 4/6 Inhibitor in Hormone Receptor-Positive, ERBB2-Negative Metastatic Breast Cancer. JAMA Netw. Open.

[27] Tarantino P., Gandini S., Nicolò E., Trillo P., Giugliano F., Zagami P., Vivanet G., Bellerba F., Trapani D., Marra A. (2022). Evolution of low HER2 expression between early and advanced-stage breast cancer. Eur. J. Cancer.

[28] Goldhirsch A., Wood W.C., Coates A.S., Gelber R.D., Thürlimann B., Senn H.J. (2011). Strategies for subtypes—Dealing with the diversity of breast cancer: Highlights of the St. Gallen International Expert Consensus on the Primary Therapy of Early Breast Cancer 2011. Ann. Oncol..

[29] Coates A.S., Winer E.P., Goldhirsch A., Gelber R.D., Gnant M., Piccart-Gebhart M., Thürlimann B., Senn H.J. (2015). Tailoring therapies—Improving the management of early breast cancer: St Gallen International Expert Consensus on the Primary Therapy of Early Breast Cancer 2015. Ann. Oncol..

[30] Allison K.H., Hammond M.E.H., Dowsett M., McKernin S.E., Carey L.A., Fitzgibbons P.L., Hayes D.F., Lakhani S.R., Chavez-MacGregor M., Perlmutter J. (2020). Estrogen and Progesterone Receptor Testing in Breast Cancer: American Society of Clinical Oncology/College of American Pathologists Guideline Update. Arch. Pathol. Lab. Med..

[31] Goldhirsch A., Winer E.P., Coates A.S., Gelber R.D., Piccart-Gebhart M., Thürlimann B., Senn H.J. (2013). Personalizing the treatment of women with early breast cancer: Highlights of the St Gallen International Expert Consensus on the Primary Therapy of Early Breast Cancer 2013. Ann. Oncol..

[32] Hammond M.E., Hayes D.F., Dowsett M., Allred D.C., Hagerty K.L., Badve S., Fitzgibbons P.L., Francis G., Goldstein N.S., Hayes M. (2010). American Society of Clinical Oncology/College of American Pathologists guideline recommendations for immunohistochemical testing of estrogen and progesterone receptors in breast cancer (unabridged version). Arch. Pathol. Lab. Med..

[33] Wolff A.C., Hammond M.E.H., Allison K.H., Harvey B.E., Mangu P.B., Bartlett J.M.S., Bilous M., Ellis I.O., Fitzgibbons P., Hanna W. (2018). Human Epidermal Growth Factor Receptor 2 Testing in Breast Cancer: American Society of Clinical Oncology/College of American Pathologists Clinical Practice Guideline Focused Update. J. Clin. Oncol..

[34] Bewick V., Cheek L., Ball J. (2004). Statistics review 12: Survival analysis. Crit. Care.

[35] Hortobagyi G.N., Stemmer S.M., Burris H.A., Yap Y.S., Sonke G.S., Paluch-Shimon S., Campone M., Blackwell K.L., André F., Winer E.P. (2016). Ribociclib as First-Line Therapy for HR-Positive, Advanced Breast Cancer. N. Engl. J. Med..

[36] Finn R.S., Martin M., Rugo H.S., Jones S., Im S.A., Gelmon K., Harbeck N., Lipatov O.N., Walshe J.M., Moulder S. (2016). Palbociclib and Letrozole in Advanced Breast Cancer. N. Engl. J. Med..

[37] Goetz M.P., Toi M., Campone M., Sohn J., Paluch-Shimon S., Huober J., Park I.H., Trédan O., Chen S.C., Manso L. (2017). MONARCH 3: Abemaciclib as Initial Therapy for Advanced Breast Cancer. J. Clin. Oncol..

[38] Sledge G.W., Toi M., Neven P., Sohn J., Inoue K., Pivot X., Burdaeva O., Okera M., Masuda N., Kaufman P.A. (2017). MONARCH 2: Abemaciclib in Combination with Fulvestrant in Women With HR+/HER2- Advanced Breast Cancer Who Had Progressed While Receiving Endocrine Therapy. J. Clin. Oncol..

[39] Slamon D.J., Neven P., Chia S., Fasching P.A., De Laurentiis M., Im S.A., Petrakova K., Bianchi G.V., Esteva F.J., Martín M. (2018). Phase III Randomized Study of Ribociclib and Fulvestrant in Hormone Receptor-Positive, Human Epidermal Growth Factor Receptor 2-Negative Advanced Breast Cancer: MONALEESA-3. J. Clin. Oncol..

[40] Cristofanilli M., Turner N.C., Bondarenko I., Ro J., Im S.A., Masuda N., Colleoni M., DeMichele A., Loi S., Verma S. (2016). Fulvestrant plus palbociclib versus fulvestrant plus placebo for treatment of hormone-receptor-positive, HER2-negative metastatic breast cancer that progressed on previous endocrine therapy (PALOMA-3): Final analysis of the multicentre, double-blind, phase 3 randomised controlled trial. Lancet Oncol..

[41] Arpino G., Bianchini G., Malorni L., Zambelli A., Puglisi F., Del Mastro L., Colleoni M., Montemurro F., Bianchi G.V., Paris I. (2022). Circulating tumor DNA (ctDNA) and serum thymidine kinase 1 activity (TKa) matched dynamics in patients (pts) with hormone receptor–positive (HR+), human epidermal growth factor 2–negative (HER2-) advanced breast cancer (ABC) treated in first-line (1L) with ribociclib (RIB) and letrozole (LET) in the BioItaLEE trial. J. Clin. Oncol..

[42] Watt A.C., Goel S. (2022). Cellular mechanisms underlying response and resistance to CDK4/6 inhibitors in the treatment of hormone receptor-positive breast cancer. Breast Cancer Res..

[43] Finn R.S., Cristofanilli M., Ettl J., Gelmon K.A., Colleoni M., Giorgetti C., Gauthier E., Liu Y., Lu D.R., Zhang Z. (2020). Treatment effect of palbociclib plus endocrine therapy by prognostic and intrinsic subtype and biomarker analysis in patients with bone-only disease: A joint analysis of PALOMA-2 and PALOMA-3 clinical trials. Breast Cancer Res. Treat..

[44] Gampenrieder S.P., Rinnerthaler G., Tinchon C., Petzer A., Balic M., Heibl S., Schmitt C., Zabernigg A.F., Egle D., Sandholzer M. (2021). Landscape of HER2-low metastatic breast cancer (MBC): Results from the Austrian AGMT_MBC-Registry. Breast Cancer Res..

[45] Hein A., Hartkopf A.D., Emons J., Lux M.P., Volz B., Taran F.A., Overkamp F., Hadji P., Tesch H., Häberle L. (2021). Prognostic effect of low-level HER2 expression in patients with clinically negative HER2 status. Eur. J. Cancer.

[46] Won H.S., Ahn J., Kim Y., Kim J.S., Song J.Y., Kim H.K., Lee J., Park H.K., Kim Y.S. (2022). Clinical significance of HER2-low expression in early breast cancer: A nationwide study from the Korean Breast Cancer Society. Breast Cancer Res..

[47] Horisawa N., Adachi Y., Takatsuka D., Nozawa K., Endo Y., Ozaki Y., Sugino K., Kataoka A., Kotani H., Yoshimura A. (2022). The frequency of low HER2 expression in breast cancer and a comparison of prognosis between patients with HER2-low and HER2-negative breast cancer by HR status. Breast Cancer.

[48] Banerji U., van Herpen C.M.L., Saura C., Thistlethwaite F., Lord S., Moreno V., Macpherson I.R., Boni V., Rolfo C., de Vries E.G.E. (2019). Trastuzumab duocarmazine in locally advanced and metastatic solid tumours and HER2-expressing breast cancer: A phase 1 dose-escalation and dose-expansion study. Lancet Oncol..

[49] Jhaveri K., Hamilton E., Loi S., Schmid P., Darilay A., Gao C., Patel G., Wrona M., Andre F. (2021). Abstract OT-03-05: Trastuzumab deruxtecan (T-DXd; DS-8201) in combination with other anticancer agents in patients with HER2-low metastatic breast cancer: A phase 1b, open-label, multicenter, dose-finding and dose-expansion study (DESTINY-Breast08). Cancer Res..

[50] Pistilli B., Wildiers H., Hamilton E.P., Ferreira A.A., Dalenc F., Vidal M., Gavilá J., Goncalves A., Murias C., Mouret-Reynier M.A. (2020). Clinical activity of MCLA-128 (zenocutuzumab) in combination with endocrine therapy (ET) in ER+/HER2-low, non-amplified metastatic breast cancer (MBC) patients (pts) with ET-resistant disease who had progressed on a CDK4/6 inhibitor (CDK4/6i). J. Clin. Oncol..

